# Large-Scale RT-qPCR Diagnostics for Seed Potato Certification

**DOI:** 10.1007/s11540-021-09491-3

**Published:** 2021-03-23

**Authors:** Olivier Schumpp, Amanda Bréchon, Justine Brodard, Brice Dupuis, Laurent Farinelli, Peter Frei, Patricia Otten, Didier Pellet

**Affiliations:** 1 Plant Protection Department, Agroscope, 1260 Nyon, Switzerland; 2 Present Address: Department of Agriculture, Food and the Marine, Plant Health Laboratory, Backweston, Celbridge, W23 X3PH Ireland; 3 Plants and Plants Products Department, Agroscope, 1260 Nyon, Switzerland; 4 Fasteris SA, Plan-les-Ouates, CH-1228 Geneva, Switzerland

**Keywords:** Certification, Deep sequencing, Molecular diagnostics, Quality control, Real-time RT-PCR, Viral disease

## Abstract

Every year, Agroscope examines nearly 300,000 tubers for the presence of viruses, as regulated for the certification of seed potatoes intended for Swiss growers. Since 2016, this examination has been performed via RT-qPCR on dormant tubers directly after harvest. This method offers fast results and eliminates the need for the use of Rindite, which is a toxic and polluting gaseous compound previously used in Switzerland to break the dormancy of seed tubers. The implementation of this molecular analytical method for the routine diagnosis of regulated viruses makes it possible to conduct additional analyses via Illumina sequencing to assess the conformity of the primers and probes used with the sequences of the different viral isolates. This form of quality control in routine diagnosis is a source of information that can answer more fundamental scientific questions related to the epidemiology of viral strains related to certification. The datasets produced in this framework can also be used to explore the diversity of rare or unknown virus species in potato crops.

**Supplementary Information:**

The online version contains supplementary material available at 10.1007/s11540-021-09491-3.

## Introduction

Several viral diseases present in all production areas of the world severely affect the yield and quality of potato production (Valkonen 2007). In Switzerland, the most damaging viruses for potato production are potato virus Y (PVY) and, to a lesser extent, potato leaf roll virus (PLRV) (Steinger et al. 2014). Both of these viruses are regulated and must not exceed an infection rate of 10% in tubers marketed as seed potatoes. These viruses are transmitted by a wide variety of aphid species (Basky 2002; Verbeek et al. 2010; Fox et al. 2017). In their winged form, these insect vectors are highly mobile and capable of efficiently spreading these viruses from one plant to another. In addition, because PVY is a so-called non-persistent virus that aphids can acquire from a diseased plant in seconds and immediately transmit to neighbouring plants, the use of insecticides is ineffective because the aphid is able to transmit the virus before it is affected by the product (Perring et al. 1999). Only the distribution of certified seedlings is effective in reducing the impact of the disease. In Switzerland, this control strategy, which was introduced in the 1960s, led to a 1.7-fold increase in yield per hectare within a few years (Keller and Bérces 1966). Based on the inoculation of indicator plants with the sap of the tuber to be tested, the labour-intensive method used when setting up the certification was replaced in 1984 by ELISA which was used to detect PVY and PLRV in seed potatoes until 2016 (Gugerli and Gehriger 1980). However, the ELISA technique is not sufficiently sensitive to detect PVY in dormant tubers immediately after harvest. Therefore, it is necessary to activate viral multiplication in the tubers to achieve ELISA-detectable virus concentrations. This activation is achieved by chemical treatment of the tubers with Rindite or by gibberellic acid-stimulated tuber culture. The first method involves manipulation of a toxic gas but allows direct ELISA analysis of the tubers, and the second method is performed on the leaves of young plants and requires a large amount of manpower as well as large greenhouse areas in which to grow the plants. In both cases, activation of viral multiplication is a lengthy process that delays the delivery of results by 4 to 8 weeks.

RT-qPCR is a molecular analysis technique that is 10^5^ to 10^7^ times more sensitive than ELISA for the detection of PVY in potato leaf extracts (Kogovsek et al. 2008), and several studies have shown that it offers a possible alternative to immunological tests for the certification process (Fox et al. 2005; Singh et al. 2012; Lacomme et al. 2015).

Based on these works, Agroscope has developed and validated an analytical method based on RT-qPCR to detect the presence of viruses from peelings collected from dormant tubers immediately after harvest. Special efforts have been made to ensure that the grinding of samples and the extraction of total RNA from tuber peelings are fast, safe, and highly scalable processes. This method has been developed and progressively validated since 2013. Since 2016, all seed potato lots produced in Switzerland have been analysed by RT-qPCR. This article details the analytical pipeline, with a processing capacity of 1000 lots in 6 weeks requiring the work of 6 to 8 people. This work also describes the implementation of a quality control step in the diagnostic process based on Illumina sequencing of RNA extracts to check for the presence of possible variants that might escape diagnostic tests, as well as the presence of rare or unknown virus diseases that are not systematically detected in seed potato lots. This control step allows the sequence of primers and probes to be modified if necessary and provides information that is of more fundamental interest, of an epidemiological nature, or related to the search for viral diversity in cultivated ecological systems.

## Materials and Methods

### Plant Material

Unless otherwise mentioned in the text, plant material was collected from seed-potato production fields before harvest. In this work, the virus control conducted as a component of the certification process was issued based on the examination of an official sample of 200 tubers per lot. RNA extraction for virus detection was performed on a 250-mg peel taken from the heel of the dormant tuber, including the stolon. After the sampling surface healed, the same tubers were subsequently treated with Rindite and analysed by ELISA. To evaluate the distribution of the virus, tubers produced by plants mechanically inoculated with PVY^NTN^ inoculum were analysed. Five tubers of the variety Charlotte, known for sensitivity to PVY^NTN^, and 5 tubers of the variety Agria, known to be more resistant to PVY^NTN^ (Dupuis et al. 2019), were sampled 8 times at different positions on the tuber and tested by RT-qPCR.

### ELISA Analysis

Double antibody sandwich (DAS)-ELISA was performed on individual sprouting tubers (Gugerli 1979; Gugerli and Gehriger 1980) with sap preparation as described by Gehriger (1986). In summary, the tubers were pierced at the base of the nascent sprout with a drill. A drop of sap was then transferred to previously coated plates with anti-PVY monoclonal antibodies (mAbs, 112511, Bioreba AG, Switzerland), and the PVY-infected tubers were detected using anti-PVY mAbs conjugate (112521, Bioreba AG, Switzerland). A tuber was considered infected if the optical density value was three times greater than the optical density of sap extracted from a healthy potato tuber tested on the same ELISA plate.

### RNA Extraction

**Fig. 1:**
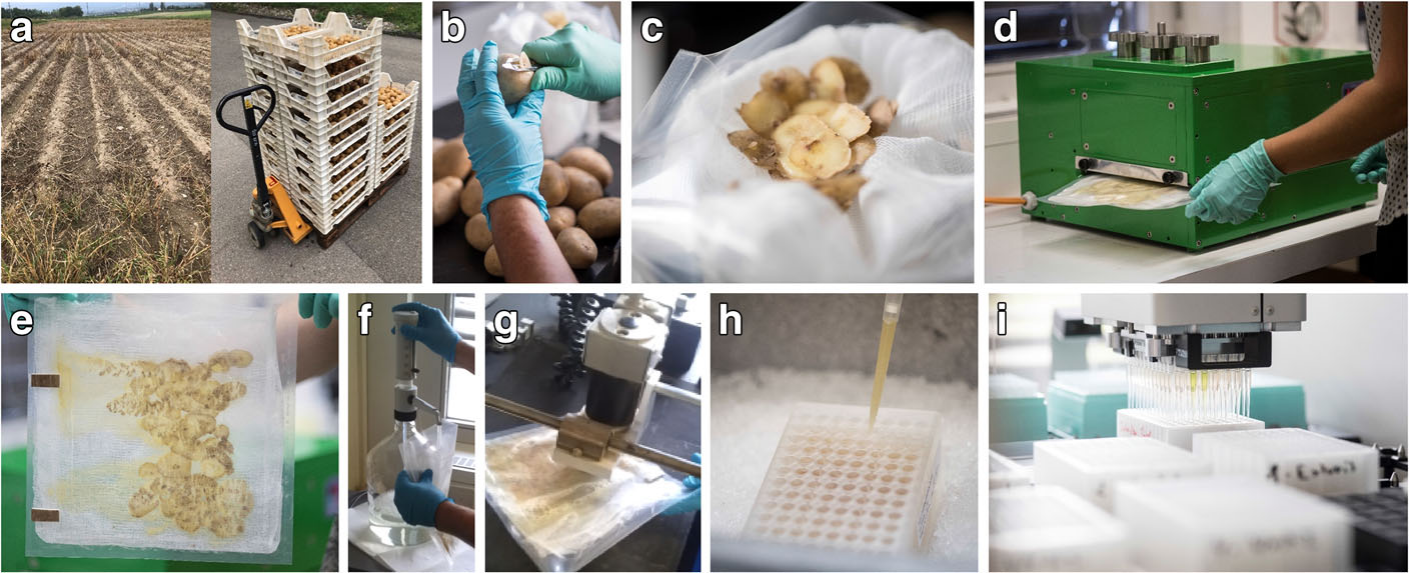
Preparation of potato homogenates in a 96-well plate format prior to RNA extraction and RT-qPCR analysis: **a** tubers are collected after top-killing and before harvest; **b**, **c** tuber peelings are assembled in a grinding bag and immediately processed through the cylinder press (**d**, **e**); **f**, **g** ground samples are immediately homogenised in stabilising buffer; **h** 1 ml of homogenate is transferred in a 96-deep-well plate; **i** further operations are performed using a liquid handling workstation

The peelings of 200 tubers from each lot were collected in groups of 25 in 8 disposable grinding bags developed and produced by Agroscope. These bags have dimensions of 24 × 28 cm, are made of PA/PE 70/120 plastic, and contain a gauze that is thermo-welded to the inner sides to allow fine grinding of the peelings by friction. Grinding of 25 peelings was performed within a few seconds using a cylinder press developed by Agroscope (Fig. 1). Fifty millilitres of stabilising buffer containing 0.02 M PBS, 5% Tween, 0.5% PVP 40, and 0.2% BSA were added after grinding, and the samples were immediately homogenised (Homex 6, Bioreba AG, Switzerland) to prevent RNA degradation. One millilitre of homogenate per bag was transferred to a 96-deep-well plate (Fig. 1) for storage at −20 °C. RNA extractions were performed in 96-well plates using the MagMAX™ Pathogen RNA/DNA Kit (Applied Biosystems, USA) with modifications. The plates were centrifuged for 3 min at 15,000 *g*, and for each well, 75 μl of supernatant were transferred to a new plate, mixed with 150 μl of Plant RNA Isolation Aid (Ambion, USA) and 300 μl of MagMAX Lysis/Binding Solution Concentrate (Invitrogen, USA), and incubated with gentle shaking for 3 min at room temperature. After centrifugation for 3 min at 15,000 *g*, 400 μl of the homogenate was transferred to a new plate containing 5 μl of MagMAX Binding Beads (Invitrogen, USA), 5 μl of Lysis Enhancer, and 400 μl of isopropanol and processed with a KingfisherFlex purification system (Thermo Fisher Scientific, USA) according to the setup and programme described in ESM_1. All manipulations were performed by a Biomek FX robotic liquid handling system (Beckman, USA) equipped with an arm with eight independent channels and a second arm equipped with a 96-channel head. The procedure described in this work allows for an extraction yield of 200 to 1000 ng of RNA in 100 μl of elution buffer (560 ± 190, *n* = 36 with Nanodrop^TM^ and 570 ± 180, *n* = 20 with Qubit^TM^). RNA samples were stored at −80 °C without a time limit in our RNA collection for subsequent analyses or verifications.

For primer development in the earliest phase of this work, cDNA was synthesised from infected potato leaf RNA using the RNeasy kit (Qiagen, USA) and processed with the iScript™ Select cDNA Synthesis Kit (Bio-Rad, USA) according to the manufacturer’s recommendations.

### Sample Bulking

The bulking strategy is based on a statistical treatment developed by Laffont et al. (2007) using the SeedCalc Excel spreadsheet available from the International Seed Testing Association webpage (https://www.seedtest.org/en/statistical-tools-for-seed-testing-_content%2D%2D-1%2D%2D3449.html). The application of statistical analysis of the potato certification scheme is described in greater detail by Lacomme et al. (2015).

**Fig. 2:**
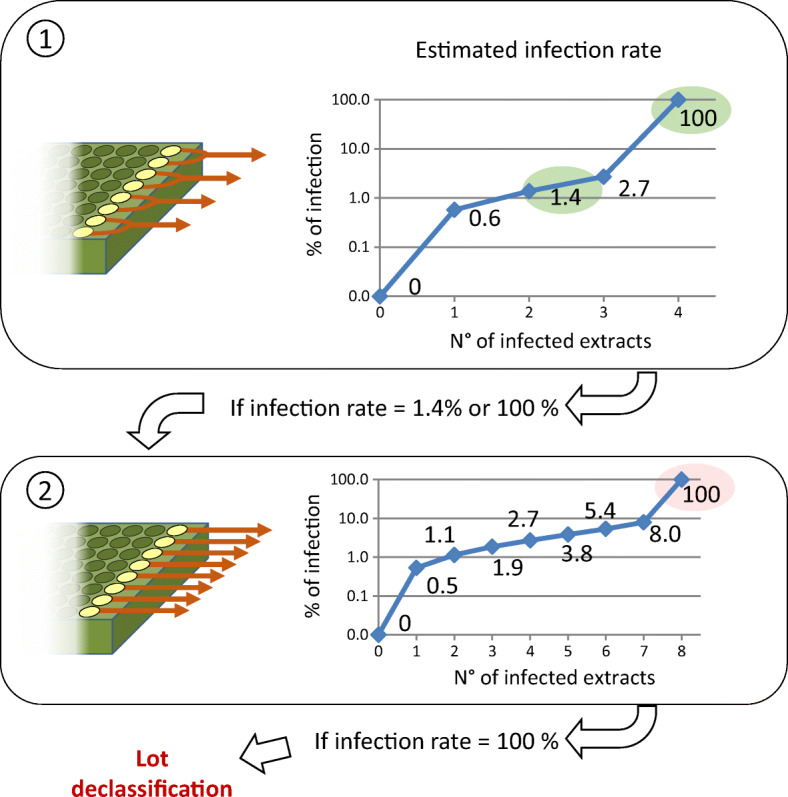
Two-stage analysis of bulked samples. Eight homogenates (1) are first pairwise assembled and analysed by RT-qPCR to estimate their infection rate using SeedCalc. Extracts from seed potato lots with an estimated infection rate of 1.4% or 100% undergo a second analysis using 8 RNA extractions to supply more detailed estimates of the infection rate

In this work, sample pooling was achieved by simultaneously grinding the 25 collected peelings in a grinding bag according to the extraction procedure described above. Each batch of 200 tubers produced 8 homogenates. In the first analysis, these 8 homogenates were pooled in pairs to produce 4 RNA extracts. RT-qPCR analysis of the 4 extracts resulted in one of the following 5 possible situations: 0, 1, 2, 3, or 4 infected lots. Based on a sample of 200 tubers, SeedCalc supplied the following 5 possible estimates for the infection rate of the lot: 0%, 0.6%, 1.4%, 2.7%, or 100%. However, these values do not include all the threshold values defined by the regulation, in particular the value of 1.1% which separates lots of the Basic seed-potato class from lots of the Certified seed-potato class (EAER_Swiss_Confederation 2020). For this reason, if a seed lot had an infection rate of 1.4% or 100% at the end of this first step, the analysis was completed by a second step in which the RNA of the 8 homogenates was extracted individually and analysed by RT-qPCR to supply estimates of infection rates of 0%, 0.5%, 1.1%, 1.9%, 2.7%, 3.8%, 5.4%, 8%, and 100% (Fig. 2). This two-step approach reduces testing costs because the majority of the lots do not require the second step.

### Primer Design

Primers and probes for Potato virus A (PVA), Potato virus M (PVM), Potato virus S (PVS), and Potato virus X (PVX) were developed as follows. Four primer sets targeting each virus were designed using Geneious R10.1 based on alignments of a selection of genomes representing 6, 10, 44, and 30 different isolates. The primer quality (i.e. ability to form cross-dimers, hairpins, and self-dimers) was controlled using NetPrimer (Premier Biosoft) and analysed with Primer-Blast at NCBI to confirm specificity. Further evaluation of specificity was performed by RT-qPCR on RNA extracts prepared from potato tubers experimentally infected with local isolates of PVY, PLRV, PVA, PVM, PVS, and PVX using SsoAdvanced™ Universal SYBR® Green Supermix (Bio-Rad, USA). For each virus, one primer set was selected based on analysis of the melting curves following RT-qPCR, and a nucleotide probe was designed using Geneious R10.1 and controlled using Netprimer (PREMIER Biosoft International, USA). For PVY and PLRV, primers and probes from Kogovsek et al. (2008) and Agindotan et al. (2007) were selected (Table 1). The sensitivity of the primer and probes sets was tested in a dilution series of RNA extracts. Primers and probes designed to verify the presence of Arabis mosaic virus (ArMV) and Strawberry latent ringspot viruses (SLRV) in RNA extracts were determined based on the alignments of Illumina reads made with Geneious R10.1 in the reference sequences identified by BLAST(n) (see below). The presence of Potato mop-top virus (PMTV) in RNA extracts was controlled by the primers mentioned by Khan et al. (2009). The forward primer was developed by Sandgren et al. (2001), but the origin of the reverse primer could not be identified.

**Table 1:** Primers and probes used in this work

Virus	Acces. Nbr.	ID	5′->3′ sequence	Fluo/quencher	Ref.	Amplicon position	Amplicon size
ArMV	-	ArMV2-G1513 Fw	ATGCGCTGGAAATTGCTATC	FAM/BHQ1	This work	1513–1602	89
		ArMV2-G1602 Rev	TGTTACGCTCATGCCCATAT				
		ArMV2-G1535 Probe	TCCTTTCGGAATGCCAGGCA				
PLRV	AY138970	PLRV 103-5 FP	AAAGCCGAAAGGTGATTAGGC	CY5/BHQ3	Agindotan et al. (2007)	5791–5859	68
		PLRV 103-5 RP	CCTGGCTACACAGTCGCGT				
		PLRV 103-5 Probe	CTCAACGCCTGCTAGAGACCGTCGAAA				
PMTV	NC_003724.1	PMTV-Fwd	CCACCCTTGGAAATGGCTGAA	Conventional RT-PCR	Sandgren et al. (2001); Khan et al. (2009)	302–861	559
		PMTV-Rv	GCCTGAGCGGTTAATTGCTAT				
PVA	NC_004039.1	PVA FP 4	GGCTATTACACCTTCAAGTTACAA	Rox/BHQ2	This work	2850–2965	115
		PVA RP 4	TGTCTTATTGCTTGAAATCTTCCA				
		PVA probe 4	TTGGAGGATTCATGGCAAGAGTTAAG				
PVM	NC_001361.2	PVM-2 FW	GCACGGAGTCTGATTATGAGGCA	FAM/BHQ1	This work	5325–5442	117
		PVM-2 REV	AACTTGTAATCCTCGATTAGATCA				
		PVM-2 Probe	TTCGAGCTGGAATTGATGAAGTAC				
PVS	NC_007289.1	PVS-4c FW	ACATTGAGCAACATCTCTTTTGAR	CY5/BHQ3	This work	7400–7527	127
		PVS-4 REV	GATCGGATTTCCATCTTGAACAGC				
		PVS-4 probe	CTTGAGCCGACCCCTGA				
PVX	NC_011620.1	PVX FP 4f	GGCCACCGTCTGAAGCTGAA	CY5.5/BHQ3	This work	6109–6284	175
		PVX RP 4f	CTGGCAAAGTCGTTGGATTG				
		PVX Probe 4f	CAAACTGCTGCCTTTGTGAAGA				
PVY	AY166866.1	PVY-Univ Fw	CATAGGAGAAACTGAGATGCCAACT	FAM/BHQ1	Kogovsek et al. (2008)	8876–8948	72
		PVY-Univ Rev	TGGCGAGGTTCCATTTTCA				
		PVY-Univ Probe	TGATGAATGGGCTTATGGTTTGGTGCA				
SLRV	-	SLRV2-G811 Fw	TCTCCTTGATGGAAATCGGG	FAM/BHQ1	This work	811–901	90
		SLRV2-G901 R	AAAGAGGGCATGAGAGTGAG				
		SLRV2-G845 Probe	GCTGTGCTTGGGATTGGGGT				

### RT-qPCR Analysis

RT-qPCR analysis was performed using three microliters of nucleic acid in a 15 μl PCR reaction (QuantiFast Pathogen RT-PCR + IC, Qiagen, USA) according to the manufacturer’s recommendations. The thermocycler programme was as follows: 1 cycle (20 min at 50 °C), 1 cycle (5 min at 95 °C), and 40 cycles (15 s at 95 °C, 45 s 60 °C).

Conventional RT-PCR for PMTV detection was performed using one microliter in a 25 μl PCR reaction with 40 U of Rnasin®, 30 U of AMV reverse transcriptase (Promega, USA), and 1.25 U of GoTaq polymerase in GoTaq buffer (Promega, USA). Primers, dNTPs and MgCl_2_ were added at concentrations of 1 μM, 0.8 mM, and 3.5 mM, respectively. The thermocycler programme was as follows: 1 cycle (45 min at 48 °C), 1 cycle (2 min at 94 °C), 35 cycles (45 s at 94 °C, 40 s at 55 °C, 90 s at 72 °C), and 1 cycle (10 min at 72 °C). Raw data obtained on a Bio-Rad CFX-96 thermal cycler (Bio-Rad, USA) were exported and analysed with LineRegPCR (Ruijter et al. 2009) to determine for each sample an individual PCR efficiency value, a Cq value, and a fluorescence amplification value. Fluorescence amplification value was established using the baseline of the amplification curve determined by LineRegPCR and the average fluorescence value of the last 5 amplification cycles. A sample was considered positive if 2 of the following 3 conditions were true: individual PCR efficiency >1.4, Delta RFU > 500 fluorescence units, and Cq < 35.

### Search for Variants That Might Escape Detection by RT-qPCR

Variant calling to control primer inclusiveness was performed each year using the RNA extracts of a subset of certified A class seed-potato lots that were previously purified for RT-qPCR analysis. Extracts were assembled in a microtube, with quality controlled using a Bioanalyzer 2100 (Agilent, USA), and used to prepare a unique sequencing library using TruSeq® Stranded Total RNA Library Preparation Kit with Ribo-Zero™ Plant treatment (Illumina, USA). The libraries were sequenced at Fasteris (Geneva, Switzerland) on an Illumina HiSeq 2500 system (Illumina, USA) in paired-end 2 × 125 nt reads.

Operations were conducted using the successive versions of Geneious regularly updated since 2016 (Geneious R9 to Geneious Prime 2020.1) according to the steps and parameters described in ESM_2. Briefly, the reads were trimmed using BBDuk and merged using BBMerge (BBMap—Bushnell B.—https://sourceforge.net/projects/bbmap/) to produce a file of merged reads and a file of reads that could not be merged. Both files were mapped on the genomes of PVY, PLRV, PVA, PVM, PVS, and PVX using the Geneious mapper. The primers and probes were annotated on the 6 alignments, and the search for variants was performed for each annotated area. The variant detection parameters included a minimum coverage of 10 reads, a frequency of at least 0.01%, and a *p* value greater than 10^-6^.

### Detection of Rare Viral Diseases

Merged reads and reads that could not be merged were assembled de novo using the Geneious assembler, and contigs were annotated using BLAST(n) and the complete NCBI RefSeq database of viral sequences (viral.1.1.genomic).

The presence of the virus was proposed based on the number of contigs that matched the viral genomes with a good *E*-value and reasonable genome coverage. Merged reads and reads that could not be merged were subsequently mapped onto the genomes of these viruses to verify the number and distribution of reads along the genomes. Primers were designed from these read alignments to verify the actual presence of these viruses in the RNA extracts by RT-qPCR and/or classic PCR and amplicon sequencing.

## Results and Discussion

### Implementation of Analytical Method

Comparisons of the RT-qPCR results with those obtained through ELISA were performed progressively on increasing numbers of lots in 2013, 2014, and 2015. After methodological adjustments and qualitative assays in 2013, a first validation step was performed on 600 tubers from six independent lots. Tubers were numbered and sampled twice for individual analysis with both detection methods (ELISA and RT-qPCR, Table 2). Both methods gave comparable results on all lots except the cv. Alexandra lot. Because 2 tubers were positive for PVY by RT-qPCR and were not detected by ELISA, the lot was downgraded from the Basic seed-potato class to the Certified seed-potato class.

**Table 2:** Analysis of individual tubers with ELISA and RT-qPCR

No. of samples	Variety	Real time RT-PCR	ELISA	Lot classification	
				Real-time RT-PCR	ELISA
1	Agria	0.0%	1.0%	Basic	Basic
2	Desiree	1.0%	0.0%	Basic	Basic
3	Alexandra	3.0%	1.0%	Certified	Basic
4	Innovator	7.0%	7.0%	Certified	Certified
5	Amandine	2.0%	2.0%	Certified	Certified
6	Nicola	1.0%	1.0%	Basic	Basic

Six lots of 100 tubers each with various infection rates were analysed by RT-qPCR and ELISA. Each tuber was sampled twice and analysed individually with ELISA and PCR. The infection tolerance threshold of the basic seed potatoes is set at 1.1%. One lot of Alexandra underwent a more severe ranking decision with RT-qPCR and was downgraded from Basic to Certified

**Fig. 3:**
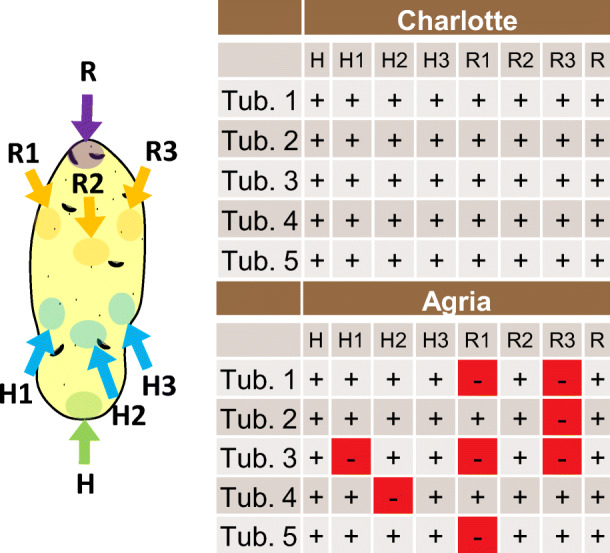
Multiple sampling on individual tubers. Five tubers from Charlotte and Agria plants mechanically infected with PVY^NTN^ were sampled 8 times at different positions, including heel-end (H) and rose-end (R). The distribution of PVY^NTN^ was homogeneous in the Charlotte variety for which all samplings were positive. However, the distribution of PVY^NTN^ particles was heterogeneous in the Agria variety in which several samplings were negative

Given that the comparison assay was based on two different samplings of the same tubers, we examined whether these variations in virus detection with each method might be explained by the distribution of the virus in the tubers, which might affect the reproducibility of the detection and thus the comparison of the two methods. The results presented in Fig. 3 show that viruses are not always homogeneously distributed in the tubers and that this feature is dependent on the cultivar and virus strain. Thus, PVY^NTN^ was present and detectable homogeneously in all 8 samples of tubers of the susceptible cultivar, whereas the presence of the virus was more random in infected tubers of the tolerant cultivar, in which 1 to 3 out of 8 samples were negative. Similar observations with different varieties were also suggested by Whitworth et al. (2012). This trend was also confirmed by our results presented in Table 2, which shows that the three susceptible cultivars Amandine, Nicola, and Innovator yielded identical results with ELISA and RT-qPCR, whereas cultivars that are tolerant to PVY such as Agria and Alexandra (Schwärzel et al. 2016) showed slightly different results among individual tubers with ELISA and RT-qPCR analysis. Therefore, the small variations observed in our validation experiments on individual tubers have a biological explanation. We conclude that the use of RT-qPCR on dormant tubers produces results that are comparable to the results obtained using ELISA on tubers for which dormancy has been lifted by the use of Rindite treatment.

The analytical method presented in this work makes it possible to quickly process a large number of seed-potato lots due to bulking a high number of samples and automation of RNA extraction and PCR assembly in 96-well plates using a high-performance liquid handling system. Validation data from comparison of this bulking strategy combined with RT-qPCR and the historical method used since 1984 in Switzerland based on ELISA analysis of individual tubers after dormancy breaking are presented in Table 3. In total, 47 lots of 200 tubers (i.e. 9400 tubers) processed in groups of 25 were analysed by RT-qPCR, and individual tubers were subsequently analysed by ELISA after the dormancy break. The two methods delivered slightly different outcomes. ELISA determines the infection percentage of a lot of 200 tubers through 200 individual analyses and supplies percent values that are multiples of 0.5%, while the statistical processing of groups of 25 using the SeedCalc application on RT-qPCR data supplies the values shown in Fig. 2 that are not multiples of 0.5. As a result, the same number of infected tubers produces slightly different numerical values depending on the analytical method, regardless of the sensitivity of the detection technique itself. Taking this observation into account, 30 lots (64%) yielded comparable results, and 17 lots (36%) showed minor differences. Of these, and because their infection rate is close to the demarcation threshold between two classes, 6 lots were certified in a different seed-potato class. Overall, RT-qPCR combined with the bulking of dormant tubers and statistical treatment produces outcomes that are highly similar to those of ELISA performed on individual tubers after dormancy break. We showed that the differences reflect biological issues rather than technical sensitivity issues.

**Table 3:** Analysis of seed potato lots with ELISA and RT-qPCR on bulked samples

Lot Number	Real-time RT-PCR		Real-time RT-PCR		% ELISA	Seed-potato class	
	First analysis		Second analysis				
	Number	%	Number	%			
						Real-time RT-PCR	ELISA
1 to 12	0/4	0.0%	-	-	0.0%	Basic	Basic
13 to 17*	0/4	0.0%	-	-	0.5%	Basic	Basic
18*	1/4	0.6%	-	-	0.0%	Basic	Basic
19 to 24	1/4	0.6%	-	-	0.5%	Basic	Basic
25 and 26	1/4	0.6%	1/8	0.5%	0.5%	Basic	Basic
27	1/4	0.6%	-	-	1.0%	Basic	Basic
28*	1/4	0.6%	3/8	1.9%	1.5%	Certified	Certified
29*	2/4	1.4%	2/8	1.1%	0.5%	Basic	Basic
30 and 31 and 33	2/4	1.4%	2/8	1.1%	1.0%	Basic	Basic
32	2/4	1.4%	3/8	1.9%	1.5%	Certified	Certified
34*	3/4	2.7%	-	-	4.0%	Certified	Certified
35	3/4	2.7%	-	-	2.5%	Certified	Certified
36	3/4	2.7%	-	-	3.0%	Certified	Certified
37	3/4	2.7%	5/8	3.9%	5.0%	Certified	Certified
38	3/4	2.7%	7/8	8.0%	6.0%	Certified	Certified
39*	4/4	> 2.7%	4/8	2.7%	2.0%	Certified	Certified
40*	4/4	> 2.7%	6/8	5.4%	7.5%	Certified	Certified
41	4/4	> 2.7%	7/8	8.0%	8.0%	Certified	Certified
42 and 43**	1/4	0.6%	-	-	1.5%	Basic	Certified
44**	2/8	1.1%	-	-	2.0%	Basic	Certified
45**	2/4	1.4%	2/8	1.1%	2.0%	Basic	Certified
46 and 47**	2/4	1.4%	2/8	1.1%	1.5%	Basic	Certified

When required, homogenates from several lots were analysed a second time by RT-qPCR (extraction 2). *Indicates lots with differences that do not affect their classification. **Indicates lots with differences that do affect their classification

In the last 5 years, this analytical method has made it possible to determine the infection rate of 900 to 1000 seed potato lots representing more than 180,000 tubers each year. The main viruses responsible for the downgrading of the lots are PVY and PLRV. PVS is also occasionally detected. PVX is even rarer and is not detected every year. Although regulated and subject to monitoring obligations, PVA and PVM have not been detected in Switzerland in recent years, regardless of the method used.

Given the average infection rates observed in seed-potato lots produced in Switzerland in recent years, a two-step analysis strategy (i.e. first analysis of 4 extracts and second analysis of 8 extracts if required) reduces analytical costs even in years in which viral incidence is high.

The strategy of bulking dormant tuber peelings reduces the number of RNA extractions to control analytical costs while retaining the ability to supply infection rate values in compliance with regulatory requirements for the classification of seed potatoes (EAER_Swiss_Confederation 2020). This method reduces the need for non-qualified personnel by approximately 40%. The analysis of dormant tubers also reduces the length of the testing campaign by 4 weeks and delivers the results more quickly, giving the national industry greater leeway to organise the seed trade.

### Control of Primers and Probes Used in Detection of Viral Strains Present in Seed Potato Lots

RT-qPCR and ELISA require the development of specific primers and antibodies for the detection of a viral strain or species, and consequently, their use is limited to the detection of characterised strains. The presence of variants in the primer target sequence or in the protein sequence targeted by the antibodies might prevent the detection of viruses and lead to false-negative diagnoses. Because new viral strains continuously emerge through genetic recombination or plant material exchanges, Agroscope performs a post-harvest grow-out assay that is applied in the following season, which allows a posteriori control of the routine analyses performed in the laboratory based on visible symptoms in the plots. This control is performed several months after the campaign and is limited to virus strains that produce visible symptoms on plant foliage.

**Fig. 4:**
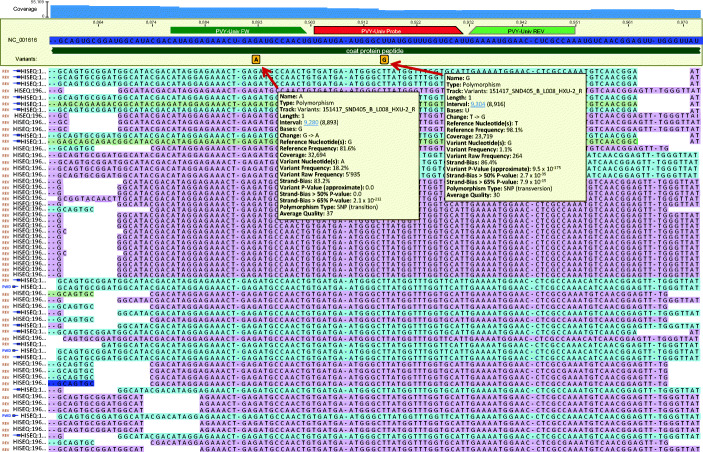
Alignment of Illumina sequencing reads on the PVY genome (Accession number AY166866.1). The positions of the primers and probe are indicated by annotations above the reference sequence. Two single-nucleotide polymorphisms were detected in the reads at positions 8893 and 8916 of the reference sequence

In contrast to the ELISA-based certification process in which tuber extracts cannot be stored or re-analysed, certification based on RT-qPCR is accompanied by the production of RNA extracts that are stored at −80 °C and are available for further analysis at a later stage. These RNA extracts are used to replace the post-harvest grow-out assay by an analysis performed immediately after the campaign to control the routine analyses conducted by RT-qPCR. A subset of extracts from class A lots are assembled, sequenced on the Illumina platform, and analysed to search for the presence of variants in the primer and probe target sequence that could prevent primer annealing and escape detection by RT-qPCR. Figure 4 shows reads produced by Illumina sequencing of an assembly of 376 RNA extracts representing 94 lots of class A seed potatoes mapped on the PVY reference genome (accession number AY166866.1). Two variants detected in the PVY-Univ Fw primer target sequence and the PVY-Univ probe target sequence at positions 8893 and 8916 were present at 18.2% and 1.1%, respectively.

This approach bypasses the problem of weakly symptomatic or asymptomatic strains that are difficult to detect in the post-harvest grow-out assay. Nevertheless, detection of a variant in the primer or probe target sequence should be approached with care and does not always indicate the need to improve the sequence of the primer or probe used in the RT-qPCR analysis. Indeed, the impact of these polymorphisms on the reliability of the diagnosis is highly variable according to the nature and position of the polymorphism on the oligonucleotide. Moreover, a weakly present variant detected only once over several years of analysis does not justify major development work. Thus, the variant detected in the PVY-Univ Fw primer at position 8893 with a value of 18.2% in 2015 (Fig. 4) and which reaches the value of 67.1% in the results of the controls carried out during the 2019 season (data not shown) clearly needs to be characterised in additional detail to check whether or not these variants escape detection with the primers and probe currently in use. This variant is the subject of ongoing work. In contrast, the polymorphism detected in the PVY-Univ Probe at position 8916 has a low frequency of 1.1% and was only detected once in 2015, and for these reasons, it has not been the subject of particular attention.

Analysis of the impact of polymorphisms detected in certification campaigns on the reliability of diagnosis by RT-qPCR and the evolution of their prevalence for all regulated viruses over the seasons will be the subject of a separate article.

### Detection of Rare Viral Diseases

Several non-regulated viruses are less frequent in Switzerland and can infect potato plants and affect the harvest quality and quantity. Among these infrequent virus infections, the Tobacco rattle virus and Potato mop-top virus are serious threats to certain sectors of the potato industry. Other viruses that are rarely identified in potato crops are of epidemiological interest, such as Tomato spotted wilt virus or Pepino mosaic virus. Finally, the increasingly widespread use of high-throughput sequencing techniques has shown the presence of an unsuspected viral diversity, the role of which remains largely not understood in most plants, including cultivated plants (Roossinck et al. 2010; Bernardo et al. 2017). Characterisation of this diversity is of more fundamental interest and is related to issues such as the interactions between viral species or the role of this diversity in ecological systems. The availability of RNA extracts produced within the framework of the analysis method presented in this work makes it possible to describe the diversity of viral sequences present in seed potato tubers to address each of these questions.

Table 4 presents the results of an analysis of Illumina sequences to search for and characterise possible infrequent viral strains in an assembly of RNA extracts. De novo assembly with the Geneious assembler produced more than 40,000 contigs. BLAST(n) analysis of these contigs using the complete NCBI RefSeq database of viral sequences identified 11 genomic fragments on which the mapping of the reads produces a large and homogeneous coverage by a high number of reads. These 11 fragments represent 7 viral species.

**Table 4:** Virus species detected by HTS on an Illumina HiSeq 2500 System (Illumina, USA) in pooled RNA extracts of 376 RNA extracts representing 18,800 tubers

Virus ID (NCBI)	Genome coverage	Number of reads	Number of infected lots	Number of infected extracts	% of infected extracts	Annotation
NC_007289.1	99.9%	111,380	8	16	4.3%	*Potato virus S, complete genome*
NC_001747.1	99.8%	58,984	10	14	3.7%	*Potato leafroll virus, complete genome*
NC_011620.1	99.6%	6420	1	2	0.5%	*Potato virus X, complete genome*
NC_001616.1	99.5%	671,011	57	131	34.8%	*Potato virus Y, complete genome*
NC_003725.1	96.5%	1224	5	10	2.7%	*Potato mop-top virus RNA 2, complete sequence*
NC_006056.1	85.0%	12,265	1	1	0.3%	*Arabis mosaic virus RNA 2, complete sequence*
NC_006964.1	71.4%	3679	1	3	0.8%	*Strawberry latent ringspot virus RNA 1, complete sequence*
NC_006057.1	59.9%	5635	1	1	0.3%	*Arabis mosaic virus RNA 1, complete sequence*
NC_003723.1	48.1%	365	5	10	2.7%	*Potato mop-top virus RNA 1, complete sequence*
NC_003724.1	40.3%	212	5	10	2.7%	*Potato mop-top virus RNA 3, complete sequence*
NC_006965.1	26.4%	1404	1	3	0.8%	*Strawberry latent ringspot virus RNA 2, complete sequence*
NC_004039.1	0%	0	0	0	0%	*Potato virus A, complete genome*
NC_001361.2	0%	0	0	0	0%	*Potato virus M, complete genome*

RT-qPCR analysis for the presence of these viruses in RNA extracts identified the number of infected RNA extracts for each virus. PVX was identified in two extracts from the same lot, PMTV was identified in ten extracts from five lots and PVS was identified in 16 extracts from eight lots. PVY and PLRV were overwhelmingly dominant and were identified in 131 extracts from 57 lots and 14 extracts from 10 lots, respectively.

Unexpectedly, ArMV and SLRV were also detected in one extract from one lot and three extracts from another lot, respectively. The detection of these two viruses that are not known to infect potato tubers illustrates the unsuspected viral diversity revealed by the use of new sequencing technologies. It is currently a question of verifying whether these viruses are indeed capable of multiplying and circulating in the plant; however, ArMV was detected a second time during the 2017 campaign, which shows that this type of marginal infection is a repeatable event. Although these viruses are not considered potato pathogens, these infections seem to be more frequent in seed potato tubers produced in Switzerland than infections by well-known potato viruses such as PVM and PVA.

Using SeedCalc, it can be deduced from the number of infected extracts that ArMV, PVX, and SLRV have infected one, two, and three tubers, respectively. Because the analysis was performed on a single batch of nucleic acids prepared from 376 RNA extracts produced from 94 lots representing 18,800 tubers, these results demonstrate the notably high sensitivity of this pipeline used in the detection of viruses in potato tubers. However, sensitivity depends on the virus species and/or genomic fragments, as illustrated by the notably low coverage of the three fragments of the PMTV genome, although they were detected by RT-qPCR in ten extracts from five batches (respectively 1/4, 3/4, 1/4, 2/4, and 3/4) representing 15 tubers, according to SeedCalc.

## Conclusions

The results obtained on a large number of different varieties infected under natural conditions show good consistency between the ELISA results of individually analysed tubers and the RT-qPCR results obtained from pooled samples. The few differences observed are minor and are attributed to occasional variations in the homogeneity of viral distribution in the tubers as shown during interactions between Agria and PVY^NTN^. This observation does not call into question the highly comparable nature of the two techniques.

RT-qPCR makes it possible to obtain results from dormant tubers, without the need for toxic and polluting chemical treatments or plant hormone treatments requiring large areas of greenhouse in which to grow the plants. The analytical pipeline presented in this article is compatible with the analysis of a large number of tubers. The time required is shortened and the security increased while maintaining costs at a level equivalent to those of ELISA due to sample bulking.

The availability of RNA extracts produced to detect viral diseases regulated within the framework of the certification process offers the potential to perform complementary Illumina sequencing. Analysis of these datasets enables detection of the presence of variants that may not be detected by RT-qPCR, thus enabling the reliability of the certification process to be inspected and guaranteeing a high level of sanitary quality control of potato seed tubers.

Furthermore, analysis of these same sequences reveals the presence of rare or as yet unidentified viruses in potatoes and offers more fundamental scientific information relevant to a better understanding of the nature and role of these rare viruses present in crops.

## Supplementary Information

ESM 1 : Reagent information and program steps for the KingfisherFlex purification system (XLSX 14 kb)

ESM 2 : Operations and parameter values used in bioinformatic analysis (DOCX 15 kb)
