# Supplementary material for: Large-Scale RT-qPCR Diagnostics for Seed Potato Certification
Source: Potato Res. 2021 Mar 23;64(4):553–69. doi: 10.1007/s11540-021-09491-3 (PMC8572825; doi:10.1007/s11540-021-09491-3)
Supplement: Supplementary file 2 — : Operations and parameter values used in bioinformatic analysis (DOCX 15 kb) [file 11540_2021_9491_MOESM2_ESM.docx]

All operations performed using **Geneious Prime® 2020.0.5**

Build 2020-01-14 05:22 Java Version 11.0.4+11 (64 bit)

**Operation 1:** “Set Paired Reads”

Parameters: Expected Distance / Insert Size 200

**Operation 2:** “Trim using BBDuk” (BBDuk Adapter/Quality Trimming Version 38.37)

Parameters:

Trim adapters Yes

Adapters All Truseq, Nextera and PhiX adapters

Trim Left hands

Kmer length 27

Maximum substitutions 1

Maximum substitutions + INDELS 0

Trim partial adapters No

Discard short reads 50 bp

**Operation 3:** “Merge paired reads” (BBMerge Paired Read Merger Version 38.37 )

Parameters: None

**Operation 4:** “Mapping” (Geneious Mapper)

Parameters: Medium sensitivity

**Operation 5:** “Assembler” (Geneious Assembler)

Parameters: Medium sensitivity

**Operation 6:** “Blast”

Parameters: DB viral.1.1.genomic

Programm Blastn

Word size 11

Gap cost 5 2

Scoring 2-3
